# Kea show no evidence of inequity aversion

**DOI:** 10.1098/rsos.160461

**Published:** 2017-03-15

**Authors:** Megan Heaney, Russell D. Gray, Alex H. Taylor

**Affiliations:** 1 School of Psychology, University of Auckland, Auckland, New Zealand; 2 Max Planck Institute for the Science of Human History, Jena, Germany; 3 Research School of the Social Sciences, Australian National University, Canberra, Australia

**Keywords:** inequity aversion, kea, cooperation

## Abstract

It has been suggested that inequity aversion is a mechanism that evolved in humans to maximize the pay-offs from engaging in cooperative tasks and to foster long-term cooperative relationships between unrelated individuals. In support of this, evidence of inequity aversion in nonhuman animals has typically been found in species that, like humans, live in complex social groups and demonstrate cooperative behaviours. We examined inequity aversion in the kea (*Nestor notabilis*), which lives in social groups but does not appear to demonstrate wild cooperative behaviours, using a classic token exchange paradigm. We compared the number of successful exchanges and the number of abandoned trials in each condition and found no evidence of an aversion to inequitable outcomes when there was a difference between reward quality or working effort required between actor and partner. We also found no evidence of inequity aversion when the subject received no reward while their partner received a low-value reward.

## 1. Introduction

Inequity aversion is defined as ‘the willingness to sacrifice material pay-offs for the sake of greater equality’ [1]. It is believed to have evolved alongside cooperation in order to maximize pay-offs and maintain cooperation between unrelated individuals [2,3]. In the laboratory, inequity aversion has been often tested in humans and animals using a token exchange paradigm. Here a subject observes a peer exchange a token for a high- or low-value reward and then trades their own token for a reward. Humans demonstrate both disadvantageous inequity aversion, where they react negatively to exchanging a token and receiving less than a peer and advantageous inequity, where they react negatively to exchanging the token and receiving more than a peer, though this second form of inequity aversion appears to be influenced by culture [1].

The first reports of disadvantageous inequity aversion in animals were reported by Brosnan & de Waal [4]. Using a token exchange procedure in which capuchin monkeys had to trade a token with the experimenter in order to receive a reward, they found that capuchins would reject a food that they had previously accepted when they witnessed another monkey receive food of higher value. They, therefore, suggested that capuchins, like humans, were averse to inequitable outcomes. Two alternative explanations have been proposed for these findings [5–9]. One is the frustration effect. This is when an animal responds negatively to receiving a low-value reward after they have previously received a high-value reward, independent of the social context it is in [5–7,9]. Another is the food expectation hypothesis, where seeing a conspecific receive and consume a high-value food reward creates the expectation that an animal will receive the same reward themselves [8].

Subsequent research examining inequity aversion in animals, most of which employs methodologies similar to Brosnan & de Waal [4], has produced mixed findings. Some studies with chimpanzees have suggested that they might be sensitive to inequity [10–12], (but see [13]), as have several studies on macaques [14,15] and one on rats. [16] Negative findings have been reported in capuchins [17,18], bonobos [8], squirrel monkeys [19,20], owl monkeys [20], marmosets [20] and orangutans [13,21]. McAuliffe *et al.* [22] reported that cotton-top tamarins generally showed little sensitivity to inequity when the required task was either effortful or effortless. However, one individual was less likely to pull in a reward tray in which the outcomes were inequitable to that of their partner when doing so was effortful. By contrast, this subject's pulling rate did not decrease in a weighted asocial control condition and a non-weighted inequity condition. Thus, there is suggestive evidence that at least one cotton-top tamarin is sensitive to inequity when a task involves effort.

Dogs were the first non-primates that appeared to exhibit inequity aversion. Range *et al*. [23] found that dogs demonstrated inequity aversion when their partner received a low-value reward and they received nothing. However, inequity aversion was not observed when their partner received a high-value reward while subjects received a reward of lower value. Based on these results, Range and colleagues suggested that dogs possess a primitive version of inequity aversion. Other non-primates have not shown evidence of inequity aversion. Carrion crows and ravens exchanged a stone less when they received a less valuable food item than their partner, and when their partner received a reward as a gift [24]. However, in line with the frustration hypothesis, exchange rates were even lower when no partner was present and the crows simply saw the high-value food reward but received the low-value one when exchanging the stone. This suggests that subjects were sensitive to the reward quality, rather than inequity. Recently, Jelbert *et al*. examined inequity aversion in New Caledonian crows using a novel stone dropping task [25]. Subjects were required to pass a stone to their partner, who would then drop it down a hole to collapse a platform containing rewards for both birds. When food was arranged on the platform so the passing crow received a smaller amount of food or a less preferred food type than their stone dropping partner, crows continued to pass the stone, suggesting this species is not averse to inequity.

One reason for the variation seen in the reaction of different species to inequity may be that only a small subset of them may have been subject to the selective pressures required for inequity aversion to evolve. Brosnan and colleagues have suggested that inequity aversion evolved alongside cooperation in humans so that individuals could form stable cooperative partnerships [2,26]. Thus only species that, like humans, are highly social and cooperative, will have evolved sensitivity to inequity. This hypothesis is supported by comparative studies which have found evidence of inequity aversion in species that are both cooperative and social, such as capuchins [4] and chimpanzees [10–12], but not in solitary species such as orangutans [13,21] or in highly social yet non-cooperative species such as squirrel monkeys [19]. However, this hypothesis is controversial as inequity aversion has not always been found in species that are both cooperative and social [17,18,20]. One way to test this hypothesis further is to examine if inequity aversion has evolved in distantly related species that are highly social but do not engage in cooperative behaviours in the wild.

Kea (*Nestor notabilis*) are endemic parrots to the South Island of New Zealand that live in complex social groups, forage extractively and exhibit high levels of play [27–29]. They do not appear to act cooperatively in the wild, though there are anecdotal reports of kea collaboratively hunting shearwater chicks. In captive conditions, this species can learn to use others as a social tool [30] and can solve a number of tasks designed to test technical intelligence [31–34]. Given the high levels of sociality in this species, and the lack of confirmed cooperative behaviours in the wild, kea are an excellent test of Brosnan's hypothesis that only highly social species that cooperate should evolve inequity aversion [2]. We, therefore, presented kea with a series of conditions designed to assess sensitivity to inequity in both reward quality and working effort, using a token exchange paradigm. We also investigated whether the absence of reward might induce inequity aversion in kea, as in dogs [23].

## 2. Material and methods

### 2.1. Subjects

We tested four sub-adult male kea, aged between 1 and 3 years (Neo, Zak, Taz and Bruce). Kea were captive bred at Willowbank Wildlife Reserve in Christchurch except for Bruce who was born in the wild and came to Willowbank as a fledgling. Subjects shared a large outdoor aviary with nine other kea. We were only able to test these four kea as they were able to monopolize the apparatus we provided and so prevent the rest of the group from interacting regularly with it. Subjects were tested in pairs within their aviary and were free to come and go from the apparatus at any time. Food and water were available ad libitum within the aviary. All aspects of this study were conducted under approval from the University of Auckland ethics committee (reference no. 001416).

### 2.2. Materials

**Figure 1.**
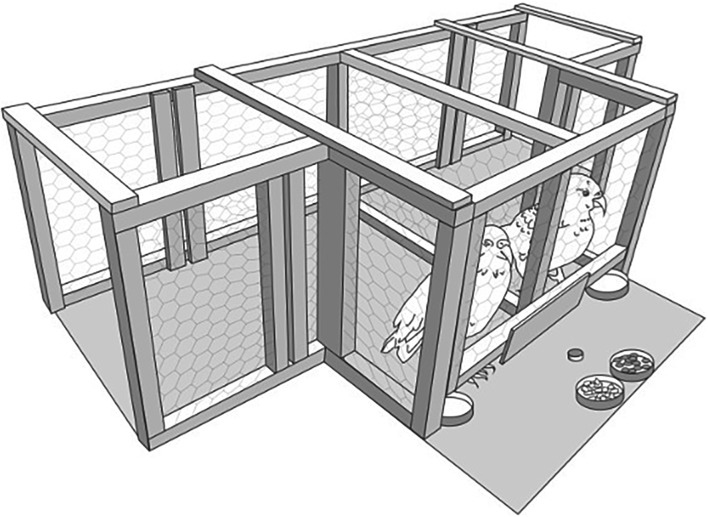
Experimental set-up and apparatus used. Rewards of each type were placed in white circular trays in the presentation area, with one reward type per tray. The experimenter offered the subject a token under the gap of the apparatus. Subjects were required to take the token and then return it to the experimenter's hand which was outstretched over the empty metal circular dishes next to the kea. The experimenter then placed the token down so that both birds could see it then gave a reward to the exchanging kea (except for trials in which the partner received a reward without having to exchange). Kea only knew which type of reward they were receiving after they had exchanged the token.

Subjects were trained and tested in a wooden apparatus (150 × 50 × 100 cm ) (figure 1) that allowed them to sit side by side and interact with the experimenter, but not interfere with each other's interactions with the experimenter and kept them relatively isolated from the rest of the group to minimize distractions. The apparatus had a wooden frame covered in chicken wire enabling subjects to see each other but not interfere with their partner's behaviour. The apparatus could be entered at two separate entry points at opposite ends of the apparatus. Once inside, neither kea could gain access to the other kea's side as the apparatus was divided down the middle with a permanent wire partition. The top of the apparatus was not covered so that subjects could exit the apparatus at any point. Pieces of wooden dowel (5 × 1 cm) were used as tokens for subjects to exchange. Tokens were attached to a thin piece of string (40 cm long) which was held by the experimenter. This was to prevent subjects from leaving the apparatus with tokens and dispersing them throughout the aviary at Willowbank. Four circular trays were positioned on the apparatus, one on the far side of each kea, within reaching distance, and two in the centre of presentation area, out of reach of the kea.

### 2.3. Procedure

#### 2.3.1. Training and familiarization

Subjects were first trained to exchange a token for a reward. Here, subjects were required to take a token offered to them by the experimenter and then return it to the experimenter's open hand in exchange for a reward. The experimenter's hand was open over a small tray which had been used in previous token exchange experiments. Once subjects had returned the token it was held up by the experimenter and placed in sight of the subject. The experimenter then delivered a reward to the subject. Training ended once each subject successfully exchanged the token for 10 trials in a row.

#### 2.3.2. Food preference test

Food preference tests were based on that used by Brosnan & de Waal [4].

*Stage 1*: This stage was to confirm that Hills Science Diet was preferred over a piece of peanut and so could be used as the high-value reward versus the low-value peanut reward. Subjects received two sessions of 10 trials where they had to choose between a piece of Hills Science Diet and a piece of peanut. The side that each reward was presented on was randomized to control for a possible side bias. Once a subject chose a reward the other reward was immediately removed. Subjects had to choose the Hills Science Diet in 80% of trials for it to be used as the high-value reward. All subjects passed this food preference test.

*Stage 2*: This checked that even though Hills Science Diet was the preferred reward subjects would still accept and consume the peanut in the absence of Hills Science Diet. To pass this stage subjects needed to accept the peanut for 10 trials in a row. Following this, subjects moved on to testing. All subjects passed this stage and no subject refused to consume the peanut on any trial.

#### 2.3.3. Experimental procedure

The experimental procedure was based on that used by Brosnan *et al.* [10]. At test the two white circular trays in the presentation area had rewards placed in them, with one reward type per tray. These rewards were out of reach of the kea but visible to them throughout testing (figure 1). To start a trial, the experimenter passed the token to a kea. The kea then had to take the token and put it into the experimenter's hand which was open over a small metal circular tray next to the kea. The experimenter then took the token, held it up and placed it in front of both kea. Following this the experimenter retrieved the appropriate reward from one of the white trays in the presentation area, held it up so it was visible to both kea and then delivered it to the exchanging bird. Partners always exchanged with the experimenter before the actor and birds knew which tray the experimenter would take a reward from to give to them after they had exchanged the token, as in past studies, e.g. [4,10,11,21,23].

Each bird was tested in six different conditions:

*Equity condition (EC)*—Baseline condition. The partner exchanged a token for a low-value food reward (peanut), then the focal kea did the same.
*Inequity condition (IC)*—Partner then focal kea exchanged, however, the partner received a high-value reward (Hills Science Diet), while the focal individual then received a low-value reward (peanut).
*Food control (FC)*—A high-value reward was held up in front of the exchanger. It was then placed back into the container it came from. The exchanging kea was then required to exchange the token. After a successful exchange it then received the low-value reward. The same method was then repeated with the other kea. This condition measured whether inequity aversion is elicited because of the mere presence of the high-value reward (individual expectation) rather than being due to seeing the partner actually consume it (social expectation).
*Free Gift (FG)*—The partner was given a low-value reward as a gift, then the focal kea exchanged and also received a low-value reward. This condition examined if kea were sensitive to working effort.
*No reward inequity condition (NRIC)*—The partner first exchanged and received a low-value reward, then the focal kea exchanged and received nothing. This condition examined whether receiving no reward while their partner receives a low-value reward might induce inequity aversion in kea, as it does in dogs [23].
*No reward partner absent (NRPA)*—Only the focal kea was present in this condition. This kea had to exchange the token but received no reward. The experimenter then held up a piece of peanut and moved it towards the empty side of the apparatus where the partnering kea would normally be. The peanut was then returned to the tray. This condition controlled for the movement of food in the NRIC condition and so assessed whether any evidence of inequity aversion in the NRIC condition was due to subjects seeing their partner receive a reward while they received nothing, or, whether a decrease in performance was because subjects simply did not receive a reward.

As in past work [10], each condition consisted of two testing sessions, each consisting of 25 trials. Thus subjects completed 50 trials of each condition. For each trial, the partner exchanged first, or in the case of the effort control (FG) condition received a reward, followed by the actor. One kea remained as actor for the entirety of both sessions of a condition, apart from conditions such as the EC and FC condition, in which both kea performed the same action and received the same reward. In this case, both birds were functionally playing the role of actor. For these conditions it was randomly decided which bird started session 1, which meant that the other bird in the dyad started session 2. The order of conditions that subjects were tested in was randomized except that no subject started with the inequity or no reward condition, nor did subjects receive two no reward conditions in a row. Before each session, subjects received five warm-up trials where they exchanged the token for a low-value reward.

For all conditions, we examined the actor's responses to the token and the food. Refusal behaviours, or other behaviours indicative of inequity aversion, such as ignoring the token, pushing the token back or refusing or ignoring the food, were scored if they met one out of several criteria, as in past research [11] (table 1). If actors did not complete an exchange within the required timeframe of 40 s (10 s to take the token and 30 s to return) they did not receive a reward. Because kea were free to come and go from the apparatus, a session was terminated if a bird left the apparatus without returning for 2 min. Behaviour was coded by two independent observers, inter-observer reliability was 100%.

**Table 1.** Classification of aversion responses.

*ignore*	experimenter offered token to actor but subject did not take token out of experimenters hand within 10 s
*refusal*	actor took token but did not return it to the experimenter within 30 s
*push back*	actor took token but pushed it back to experimenter
*food ignore*	actor did not take food offered by experimenter within 10 s
*food refusal*	actor took food but did not consume within 30 s

### 2.4. Dyad formation

Dyads were chosen based on pairs that had worked together in previous experiments. The dyads remained the same and as in Range *et al*. [23], one subject completed all conditions before the other and the order in which subjects completed sessions was pseudorandomized. However, there were two conditions (EC, FC) in which the actions and rewards were the same for both birds. Therefore, as in Brosnan *et al*. [21], in these conditions both birds served as the role of subject. So while one bird completed testing first, when it was their partners turn, their partner had already completed the former two conditions. Condition order is reported in electronic supplementary material, table S1.

## 3. Results

A non-parametric Friedman's test was conducted to examine whether there were any differences in the percentage of successful exchanges between the four conditions in which actors always received a reward (EC, IC, FC, FG). There was no significant differences between conditions (*χ*^2^ = 2.00, *p* = 0.57). This indicates that subjects were not sensitive to inequity in regards to reward quality or the working effort of their partner. There were also no other aversion behaviours, such as ignoring the token, pushing the token back or refusing or ignoring the food, exhibited by any birds in any of these four conditions in which the actor received a reward (EC, IC, FC and FG).

**Figure 2.**
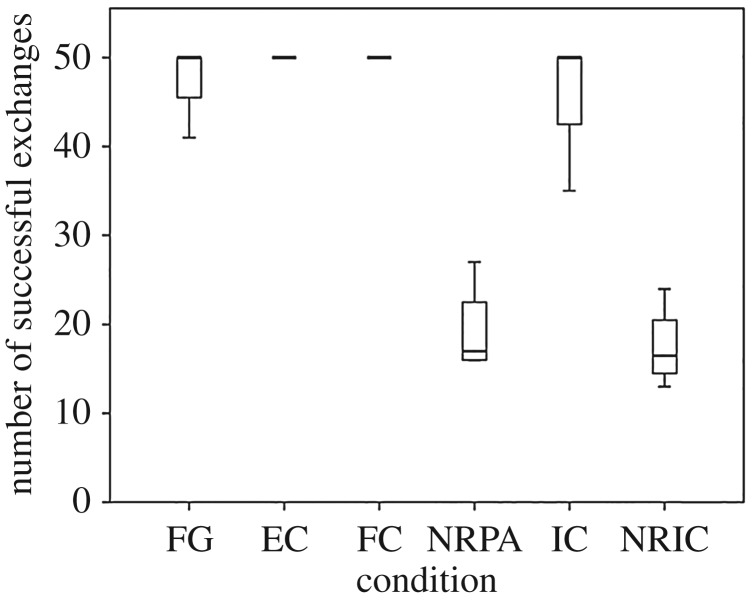
Box plots showing the average number of time actors successfully exchanged the token with the experimenter for each condition (out of a maximum of 50 trials). Boxes represent the interquartile range, lines within boxes represent median values and whiskers represent the 5th and 95th percentiles.

A non-parametric Friedman's test were then conducted to examine whether there was a difference between the baseline equity test and the two no reward conditions (partner absent and partner present). A significant difference was found between these conditions (*χ*^2^ = 6.53, *p* = 0.38), suggesting that token exchanges dropped significantly between the baseline equity test and one or both of the no reward conditions (NRPA and NRIC, figure 2). We then ran a Wilcoxon signed-rank test between the two no reward conditions to analyse whether the drop in exchanges was due to subjects witnessing their partner receive a reward while they received nothing or whether it was simply due to not receiving a reward themselves. No significant difference between the number of successful exchanges was found (*Z *= −0.535 *p* = 0.593). Therefore, the fact that subjects did not receive a reward themselves, regardless of whether they witnessed their partner receive a reward, explains why performance significantly dropped in these conditions.

**Figure 3.**
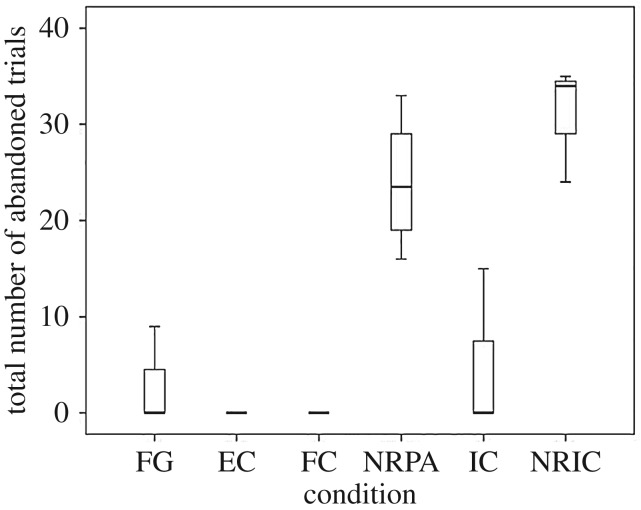
Box plots showing the number of abandoned trials for each condition. Boxes represent the interquartile range, lines within boxes represent median values and whiskers represent the 5th and 95th percentiles.

We next analysed the total number of trials that kea abandoned in each condition (figure 3). A non-parametric Friedman's test revealed that there were no significant differences in the number of abandoned trials between the four reward conditions (EC, IC, FC, FG; *χ*^2^ = 2, *p* = 0.572). In the two no reward conditions, subjects abandoned more trials in the partner present, no reward condition (mean = 29.25 out of 50 possible trials s.d. = 5.50), compared with the no reward, partner absent condition (mean = 24.00, s.d. = 7.07). However, a Wilcoxon signed-rank test only revealed a non-significant trend for a difference in the number of abandoned trials between these two conditions (*Z *= −1.841, *p* = 0.066). The rate of aversive behaviours per trial was also not significantly different between the two no reward conditions (*Z* = −1.069, *p* = 0.285; figure 3).

## 4. Discussion

Kea did not pass the token less often when their partner received a high-quality food item and they received a low-value one, nor did they show aversion behaviours in response to this inequitable situation. They also did not pass the token less when their partner was given a low-value reward for free and they had to exchange a token to receive the same reward. Kea did pass the token significantly less when their partner exchanged a token for a reward, and they exchanged and received nothing. However, the pass rate was not significantly higher in the asocial control condition, where the kea passed and did not receive a reward without a partner present. This suggests that the drop in token exchange rates was not due to the kea seeing the partner get a reward while they receive nothing, but simply because they were not being rewarded themselves for token exchange.

These results are in contrast with those found with other species. Kea showed no evidence of reacting to inequity in food value, as has been claimed in capuchins, rats, macaques and chimpanzees [4,11,14–16], nor did they react to inequity in effort, as has been suggested in dogs and cotton-top tamarins [22,23]. They also did not react to manipulations in their expectation of food type, as seen in crows and ravens [24]. Instead kea reacted like a wide range of other species, including bonobos [8], squirrel monkeys [19], orangutans [13,21] owl monkeys [20], marmosets [20], cleaner fish [35] and New Caledonian crows [25], in showing no significant reaction to inequitable situations.

Given our small sample size further research is clearly required to confirm that kea are insensitive to inequity, particularly given recent research that has failed to find evidence of inequity aversion in populations of some primate species [13,18]. However, our findings are in line with the hypothesis that inequity aversion evolved only in highly social and cooperative species to maintain cooperative bonds between unrelated individuals [2,26]. Kea are highly social and engage in high levels of play, but do not appear to cooperate in the wild. They, therefore, should not have evolved aversion to inequity, a prediction in line with the results reported here.

## Supplementary Material

ESM Data on condition order, exchanges, abandoned trials and refusals

## Data accessibility

Data are available in the electronic supplementary material.
