# Supplementary material for: Kea show no evidence of inequity aversion
Source: R Soc Open Sci. 2017 Mar 15;4(3):160461. doi: 10.1098/rsos.160461 (PMC5383808; doi:10.1098/rsos.160461)
Supplement: ESM Data on condition order, exchanges, abandoned trials and refusals [file rsos160461supp1.docx]

**Supplementary materials**

Table S1:

Overview of order of conditions received by each subject

| **Subject** | **Condition 1** | **Condition 2** | **Condition 3** | **Condition 4** | **Condition 5** | **Condition 6** |
| --- | --- | --- | --- | --- | --- | --- |
| **Neo** | IC | FG | NRIC | FC | EC | NRPA |
| **Zak** | EC | FC | NRPA | FG | NRIC | IC |
| **Taz** | IC | NRPA | FG | NRIC | EC | FC |
| **Bruce** | EC | FC | NRIC | IC | NRPA | FG |

Table S2: Number of successful exchanges for each bird in each condition (out of 50 possible trials)

| **Subject** | **EC** | **IC** | **FC** | **FG** | **NRIC** | **NRPA** |
| --- | --- | --- | --- | --- | --- | --- |
| ***Neo*** | 50 | 50 | 50 | 50 | 24 | 16 |
| ***Zak*** | 50 | 35 | 50 | 50 | 16 | 16 |
| ***Kati*** | 50 | 50 | 50 | 50 | 17 | 27 |
| ***Taz*** | 50 | 50 | 50 | 41 | 13 | 18 |

Table S3 Number of abandoned trials for each bird in each condition

| **Subject** | **EC** | **IC** | **FC** | **FG** | **NRIC** | **NRPA** |
| --- | --- | --- | --- | --- | --- | --- |
| ***Neo*** | 0 | 0 | 0 | 0 | 24 | 22 |
| ***Zak*** | 0 | 15 | 0 | 0 | 34 | 33 |
| ***Kati*** | 0 | 0 | 0 | 0 | 25 | 16 |
| ***Taz*** | 0 | 0 | 0 | 9 | 34 | 25 |

Table S4 Number of refusals for each bird in each condition

| **Subject** | **EC** | **IC** | **FC** | **FG** | **NRIC** | **NRPA** |
| --- | --- | --- | --- | --- | --- | --- |
| ***Neo*** | 0 | 0 | 0 | 0 | 1 push back  1 ignore | 4 refusals  8 ignore |
| ***Zak*** | 0 | 1 | 0 | 0 | 1 ignore | 1 refusal |
| ***Kati*** | 0 | 0 | 0 | 0 | 6 refusals  2 ignore | 6 pushback  1 refusal |
| ***Taz*** | 0 | 0 | 0 | 1 | 3 ignore | 5 pushback  1 ignore  1 refusal |
